# Putting the Mess in Order: *Aspergillus welwitschiae* (and Not *A. niger*) Is the Etiological Agent of Sisal Bole Rot Disease in Brazil

**DOI:** 10.3389/fmicb.2018.01227

**Published:** 2018-06-11

**Authors:** Elizabeth A. A. Duarte, Caroline L. Damasceno, Thiago A. S. de Oliveira, Leonardo de Oliveira Barbosa, Fabiano M. Martins, Jurema Rosa de Queiroz Silva, Thais E. F. de Lima, Rafael M. da Silva, Rodrigo B. Kato, Dener E. Bortolini, Vasco Azevedo, Aristóteles Góes-Neto, Ana C. F. Soares

**Affiliations:** ^1^Center of Agricultural, Environmental and Biological Sciences, Federal University of Reconcavo of Bahia, Cruz das Almas, Brazil; ^2^Graduate Program in Biotechnology (PPGBiotec), State University of Feira of Santana, Feira de Santana, Brazil; ^3^Department of Biological Sciences, State University of Feira of Santana, Feira de Santana, Brazil; ^4^Department of Microbiology, Institute of Biological Sciences, Federal University of Minas Gerais, Belo Horizonte, Brazil

**Keywords:** black aspergilli, *Agave sisalana*, red rot, microbial interaction, Aspergillosis

## Abstract

Approximately 75% of the worldwide production of hard natural fibers originates from sisal, an industrial crop from arid and semiarid tropical regions. Brazil is the world's largest producer of sisal fiber, accounting for more than 40% of the worldwide production, and sisal bole rot disease has been the main phytosanitary problem of this crop. All previous studies reporting *Aspergillus niger* as the causal agent of the disease were based on the morphological features of fungal isolates from infected plant tissues in pure cultures. Black aspergilli are one of the most complex and difficult groups to classify and identify. Therefore, we performed an integrative analysis of this disease based on the isolation of black aspergilli from the endospheres and soils in the root zones of symptomatic adult plants, *in vivo* pathogenicity tests, histopathology of symptomatic plants, and molecular phylogeny and worldwide genetic variability of the causal agent. All sisal isolates were pathogenic and unequivocally produced symptoms of bole rot disease in healthy plants. In all tree-based phylogenetic methods used, a monophyletic group formed by *A. welwitschiae* along with all sisal isolates was retrieved. Ten *A. welwitschiae* haplotypes have been identified in the world, and three occur in the largest sisal-producing area. Most of the isolates are from a unique haplotype, present in only the sisal-producing region. *A. welwitschiae* destroyed parenchymatic and vascular cylinder cells and induced the necrosis of internal stem tissues. Therefore, sisal bole disease is probably the consequence of a saprotrophic fungus that opportunistically invades sisal plants and behaves as a typical necrotrophic pathogen.

## Introduction

Sisal (*Agave sisalana* Perrine), also known as sisal hemp, is a perennial monocotyledonous succulent plant of the family Asparagaceae that has thick leaves in a basal rosette (aerial stem) (WCSP-World Checklist of Selected Plant Families in the Catalogue of Life, 2016) and originates in the semiarid and arid areas of Mexico (García-Mendoza, 2002; Coleman-Derr et al., 2016). Sisal has several morphological and physiological characteristics that make it well adapted to arid and semiarid regions worldwide (Mielenz et al., 2015; Stewart, 2015). Like other *Agave* species, sisal utilizes the Crassulacean acid metabolism (CAM) pathway for CO_2_ fixation, with stomatal opening during the night due to less evapotranspiration and better water efficiency at the lower night temperatures (Abraham et al., 2016). In addition, the leaves have a thick, waxy cuticle and are arranged in a spiral shape around the stem, forming a rosette, which favors water retention (Ortiz and Van der Meer, 2006). These leaf morphological features also give protection against insects and pathogenic microorganisms (Silva and Beltrão, 1999; Neto and Martins, 2012). Sisal is a monocarpic plant that forms an inflorescence after six to nine years and subsequently dies (Asfaw, 2011). Its reproduction is mainly asexual via suckers originating from the rhizomes (subterraneous stems) and bulbils produced in the inflorescence (Moreira et al., 1999).

The important physiological features of *Agave* species as CAM plants, which have thick, waxy leaf cuticles and stomata and roots adapted to drought, have been pointed out by several authors (Corbin et al., 2015; Yang et al., 2015; Davis et al., 2017). These authors reinforce the importance of these plants for several uses, such as the production of bioenergy in semiarid and arid environments, which are considered marginal agricultural lands, in the USA, Australia, Mexico, Brazil, African countries and many other tropical parts of the word.

The main economic application of sisal is in the textile industry, as approximately 75% of all hard natural fibers are produced from sisal (Parsons and Darling, 2000; Rousso, 2010). These fibers are used for making agricultural baler twine and to make carpets, rugs, sacks, yarns, ropes and other cordage (Müssig, 2010). Sisal has also been studied as a medicinal plant (Chen et al., 2009; Debnath et al., 2010), as feedstock for bioenergy (Yang et al., 2015; Davis et al., 2017), as a source of saponins (Sidana et al., 2016) and for producing biocomposites in substitution of glass, carbon and polymeric (plastics) fibers (Scopel et al., 2013). Furthermore, residues from the decortication of the leaves can be used for animal feed (Faria et al., 2008) and have been studied for their nematicidal (Jesus et al., 2014; Damasceno et al., 2015), antimicrobial (Santos et al., 2009), and insecticidal properties (Sousa et al., 2014).

Sisal plants were first exported from the region of Yucatan in Mexico to southern Florida (USA) in 1834, and they were introduced to Tanzania (former Tanganyika, Africa) in 1893 (Kimaro et al., 1994). All sisal plants distributed in several tropical and subtropical countries are suggested to have originated from these Yucatan-exported plants (Medina, 1954). This plant was introduced to Brazil from bulbils that were brought from Florida (USA) in 1903 by the entrepreneur Horácio Urpia Júnior, who initiated the first commercial plantation and production of sisal fibers in this country in the municipality of Maragogipe in the state of Bahia (Pinto, 1969). At the worldwide level, Angola, Kenya, Madagascar, Mozambique, South Africa, and Tanzania (in Africa); China, Indonesia, and Thailand (in Asia); and Mexico, Cuba, and Haiti (in North and Central America) produce fibers from sisal or other *Agave* species, with a worldwide production of 300,000 tons/year (Food and Agriculture Organization, 2015). Brazil is the world's largest producer of sisal fiber, accounting for more than 40% of the world production (Food and Agriculture Organization, 2015), and the state of Bahia claims approximately 95% of the Brazilian sisal production (IBGE-Instituto Brasileiro de Geografia e Estatística, 2015). This crop has become socially and economically important in the semiarid region of Brazil because of the number of jobs generated in its production chain. Furthermore, sisal has become the main agricultural product for small family-based farming systems in very poor regions of the state of Bahia (Silva and Beltrão, 1999; CONAB Companhia Nacional de Abastecimento, 2016).

Sisal bole rot, also known as sisal red rot disease, has been the main phytosanitary problem in sisal plantations in Brazil, with 100% prevalence and an average incidence of 35% in sisal-producing areas (Abreu, 2010). This disease, which can lead to plant death, was initially detected in sisal plantations in Tanzania in the 1930's (Wallace, 1937), but it was only described approximately 20 years later by Wallace and Dieckmahns (1952). In their seminal paper, these authors pointed out that the disease was caused by *Aspergillus niger*, a common and widespread soil saprotrophic fungi, and some mechanical injury to the plant (caused by human management and/or insects) is required for the infection to occur. In an extensive review of sisal, Medina (1954) reported a description by Machado (1951) of a sisal light-brown stem base rot in the state of Paraiba, Brazil, which is the first citation of this disease in Brazil. Medina (1954) also suggested that the sisal red rot described in Venezuela by Ciferri (1951) and the sisal stem rot in Anjouan Island (Comores, Africa) reported by Crétenet and De Raucourt (1959) were all descriptions of the same disease.

In Brazil, sisal bole rot was first attributed to *Botryodiplodia theobromae* (Lima et al., 1998) and later to *A. niger*, with disease incidences varying from 5 to 40% (Coutinho et al., 2006). Pathogenicity tests and the satisfaction of Koch's postulates were reported, and symptoms were observed in only wounded inoculated plants under greenhouse conditions. These symptoms were described as yellowish leaves (external symptoms) and brown internal tissue surrounded by reddish tissue inside sisal aerial stem (internal symptoms) (Coutinho et al., 2006). Santos et al. (2014) reported that *A. niger, Aspergillus brasiliensis*, and *Aspergillus tubingensis*, isolated from the soils of sisal-producing areas, were able to cause this disease in sisal plants inoculated under greenhouse conditions. Nevertheless, these authors pointed out that only *A. niger* was isolated from field symptomatic plants, and the epidemiological significance of the other two *Aspergillus* species remains unknown.

Since the first description of the disease (Wallace, 1937), assignment of the etiological agent has been controversial, and distinct microorganisms (even from distinct kingdoms), such as *Pythium aphanidermatum* (Chromista, Oomycota) (Bock, 1965), *Lasiodiplodia theobromae* (Fungi, Ascomycota: Dothidiomycetes) (Lima et al., 1998) and *A. niger* (Fungi, Ascomycota: Eurotiomycetes), with the latter being the most common (Wallace and Dieckmahns, 1952; Coutinho et al., 2006), have been associated with this disease. However, all these studies that identified the causal agent of the disease as *A. niger* were based on solely macro- and micromorphological features of the fungal isolates from plant infected tissues in pure culture.

Black aspergilli (*Aspergillus* section *Nigri*) are one of the most complex, confusing, and difficult groups to classify and identify (Varga et al., 2011). This taxonomic group includes six different clades (Samson et al., 2014) and 26 distinctly recognized species (Ismail, 2017). Thus, species of *Aspergillus* section *Nigri* are morphologically very similar and, in many cases, phenotypically indistinguishable and can only be reliably identified by the calmodulin (CaM) gene and not by the primary (nrITS) fungal DNA barcode (Susca et al., 2016). Moreover, none of the aforementioned works performed a detailed histopathological analysis of the disease.

Therefore, along with a thorough reappraisal of the sisal bole rot, we aimed to perform an integrative study of this fungal disease comprising the following phytopathological, microbiological and molecular analyses: (i) the isolation of black aspergilli in the endosphere (roots/stems/leaves) and corresponding soils in the root zones of symptomatic adult sisal plants in commercial plantations; (ii) *in vivo* pathogenicity tests, including the time-course of the infection with black aspergilli isolated from plant endospheres and the corresponding soils in the root zones; (iii) the histopathology of the symptomatic plants; and (iv) a comprehensive molecular phylogeny and world-level genetic variability of the etiological agent of the sisal bole rot using the CaM gene.

## Materials and methods

### Field sampling of endosphere tissues and corresponding soils in the root zones of adult sisal plants from commercial plantations

The fieldwork was performed in commercial plantations in the rural zone of three municipalities of the main sisal-producing area of the world, the Brazilian semiarid region of the state of Bahia with the typical BSh climate in the Köppen system: (i) Conceição do Coité (11°40′0″S, 39°20′0″W), with a mean annual temperature of 22.3°C, a mean annual rainfall of 585 mm, and a mean elevation of 428 m; (ii) São Domingos (11°27′56″S, 39°31′34″W), with a mean annual temperature of 23.4°C, a mean annual rainfall of 521 mm, and a mean elevation of 290 m; and (iii) Retirolândia (11°28′46″S, 39°24′58″W), with a mean annual temperature of 23.1°C, a mean annual rainfall of 525 mm, and a mean elevation of 315 m (INPE – Instituto Nacional de Pesquisas Espaciais., 2017). The soil characteristics of each site are presented in Supplementary Table 1.

Samples of leaf, stem and root tissues from adult plant specimens of sisal (*Agave sisalana* Perrine) and the corresponding soil in the root zone were collected in the dry and rainy seasons. For each study site, four plant specimens exhibiting the typical symptoms of sisal bole rot (chlorotic and wilted leaves associated with a reddish color in stem tissue and at the base of the leaves) were selected randomly. Two samples of each tissue, leaf, stem (red rotten margin), and root as well as the soil from all plant specimens were collected. The soil samples were collected around the sisal plant root zone along a 0–20 cm deep and were sieved through a 2 mm mesh. Tissue and soil samples of all plant specimens were stored at 4°C until processing.

### Isolation, maintenance, and preservation of sisal endospheres and root zone soil-associated black aspergilli

The intact samples of plant tissues (roots, stems and leaves) were washed with sterilized distilled water, and the fragments were aseptically removed from them using sterilized scalpels. Six 25 mm^2^ fragments (two from each region of the samples: apical, median and basal) were removed from all the tissue samples. The fragments were surface-sterilized via successive dipping in 70% ethanol (1 min), 2% sodium hypochlorite (1 min), and 70% ethanol (30 s), followed by washing with sterile distilled water (1 min) three times in a laminar flow-hood (Pereira et al., 1993). To test the effectiveness of the surface sterilization, 100 ml of the water used during the final rinse was plated onto potato dextrose agar (PDA) to test for epiphytic microbial contaminants. All the tissue fragments were plated onto PDA with chloramphenicol (50 mgL^−1^), incubated at 25°C and daily examined for up to 15 days. The isolation of root zone soil-associated fungi was performed from a serial dilution of 10 g of soil in the root zone in 90 mL of sterilized saline solution (NaCl at 0.85%). Decimal aliquots (1:10) of the original solution were plated onto PDA with NaCl at 6% (Dhingra and Sinclair, 1994), incubated at 25°C and examined at every 3 days for up to 15 days.

Colony-forming units (CFUs), with the typical morphological features of black aspergilli, were subcultured by transferring a colony fragment to new sterilized PDA medium. The isolated black aspergilli CFUs were then characterized by macro- and micromorphology and further grown in pure culture. Five replicates of all the fungal isolates were preserved in sterile distilled water (Castellani, 1967), cryopreserved in 20% glycerol, and further deposited in the Culture Collection of Microorganisms of Bahia (CCMB) (Table 1).

**Table 1:** Plant endosphere- and rhizosphere-associated black aspergilli isolated from symptomatic plants in the study areas.

**Number**	**Isolate code**	**Host**	**Plant/environment compartment**	**Access n°. in CCMB**	**Access n°. in NCBI**
1	RSDI	*Agave sisalana*	Root	CCMB709	MG322287
2	RSDII	*Agave sisalana*	Root	CMB703	MG322289
3	RRI	*Agave sisalana*	Root	CCMB668	MG322286
4	RRII	*Agave sisalana*	Root	CCMB670	MG322290
5	RCI	*Agave sisalana*	Root	CCMB706	MG322272
6	RCII	*Agave sisalana*	Root	CMB662	MG322277
7	CSDI	*Agave sisalana*	Stem	CMB663	MG322270
8	CSDII	*Agave sisalana*	Stem	CCMB710	MG322283
9	CRI	*Agave sisalana*	Stem	CCMB678	MG322280
10	CRII	*Agave sisalana*	Stem	CCMB707	MG322291
11	CCI	*Agave sisalana*	Stem	CCMB674	MG322278
12	CCII	*Agave sisalana*	Stem	CCMB672	MG322275
13	FSDI	*Agave sisalana*	Leaf	CCMB665	MG322292
14	FSDII	*Agave sisalana*	Leaf	CCMB669	MG322285
15	FRI	*Agave sisalana*	Leaf	CCMB666	MG322281
16	FRII	*Agave sisalana*	Leaf	CCMB676	MG322276
17	FCI	*Agave sisalana*	Leaf	CCMB704	MG322279
18	FCII	*Agave sisalana*	Leaf	CCMB667	MG322284
19	SSDI	*Agave sisalana*	Root zone soil-associated	CCMB675	MG322282
20	SSDII	*Agave sisalana*	Root zone soil-associated	CCMB677	MG322288
21	SRI	*Agave sisalana*	Root zone soil-associated	CCMB708	MG322273
22	SRII	*Agave sisalana*	Root zone soil-associated	CCMB673	MG322271
23	SCI	*Agave sisalana*	Root zone soil-associated	CCMB671	MG322274
24	SCII	*Agave sisalana*	Root zone soil-associated	CCMB705	MG322269

### Koch's postulates and pathogenicity tests on plant endosphere- and root zone soil-associated black aspergilli isolated from symptomatic plants under greenhouse conditions in sisal bulbils

Pathogenicity tests on black aspergillus isolates from the plant endosphere (roots, stems, leaves) and corresponding soil in the root zone were carried out in sisal bulbils (approximately 4 months old, 30-cm in height, 8–10 leaves) in plastic pots with 1 kg of soil under greenhouse conditions. Fungal isolates were cultured in 9-cm Petri dishes with PDA and incubated for 5 days with a 12-h photoperiod at 25 ± 1°C. Inocula were prepared by pouring a solution of 0.03% Tween 20 in sterilized distilled water into the plates with the fungal cultures and scraping the colonies with a sterile inoculating loop. The colony suspensions were filtered through sterile cheesecloth, and spore suspensions were counted with a Neubauer chamber under a light microscope and adjusted with sterile water to the final concentration of 1 × 10^6^ spores ml^−1^. The same solution without fungal inoculum was used to set the control plants. Healthy (asymptomatic) sisal bulbils (30 cm height) were inoculated with an inoculum (50 μl of spore suspension) deposition into three micro-wounds (3-mm depth, performed with a hypodermic needle) in the stem tissue. Control plants were treated in the micro-wounded stem with the 0.03% Tween 20 solution without inoculum. The inoculated and control plants were maintained under greenhouse conditions with irrigation every 3 days. This experiment was carried out in a completely randomized design (CRD) with 10 replicates. All plants were observed daily for symptoms of bole rot disease for 30 days. After observation of external symptoms (leaf wilting and/or red rot symptoms at the leaf base region close to the stem), the plants were cut vertically through the stem tissue for the observation of internal symptoms.

To confirm the pathogenicity of the black aspergillus isolates, re-isolations from lesions in sisal stems were carried out immediately after symptom observation. Small fragments excised from the stems were surface-sterilized via successive dipping in 70% ethanol (1 min) and 1% sodium hypochlorite (1 min), followed by washing with sterile distilled water 3 times. Excess water was removed using sterilized filter paper (Whatman no. 1). The fragments were inoculated in 9-cm Petri dishes with PDA and incubated for 5 days with a 12-h photoperiod at 25 ± 1°C. The morphologies of the fungal colonies were compared with the initial isolates, and the re-isolated strains were further sequenced to verify their molecular identity and re-inoculated in healthy sisal bulbils to confirm Koch's postulates.

### Anatomical and histological analyses of the infected stems of adult sisal plants from commercial plantations

Stem samples of five adult specimens with visible symptoms of bole rot disease were collected from the sisal-producing areas of Conceição do Coité, Bahia, Brazil. These samples were also used for isolating black aspergilli for molecular identification and pathogenicity tests. The stem tissue samples were vacuum-fixed in FNT (buffered neutral formalin: phosphate buffer and formalin, 9:1 v/v) for 48 h in a desiccator (Lillie, 1965) and further conserved in 70% ethanol. The fixed samples were then dehydrated in a graded ethanol series and embedded in 2-hydroxymetyl methacrylate (Historesin, Leica) according to Meira and Martins (2003). Serial transversal and longitudinal thin sections (5–12 μm) were obtained using a semi-motorized rotary microtome (Leica RM2245). The thin sections were stained with toluidine blue (pH 4.4) (Steer, 1982) and double-stained with toluidine blue and basic fuchsin (Junqueira, 1990) to observe and distinguish the fungal structures in plant tissues. The slides were mounted with synthetic resin (Permount/Fisher) and photographed using an Olympus BX51 photomicroscope equipped with a digital photographic camera (Olympus A330). Figure scales were obtained via the projection of a photographed/digitized micrometric slide in the same optical conditions of the photos.

### Morphological characterization of sisal endosphere- and root zone soil-associated black aspergilli

Morphology was analyzed according to the criteria of Samson et al. (2014). Czapek yeast extract agar (CYA), malt extract agar (MEA), Czapek agar (CZA), and oatmeal agar (OA) were used for macromorphological characterization. The isolates were inoculated at three points on each plate of each medium and incubated at 25, 30, or 37°C in the dark for 7 days, and the plates were unwrapped to allow for sufficient aeration. The macromorphological features used for characterizing the species included the colony growth rate, texture, degree of sporulation, production of sclerotia, mycelium color, sporulation, soluble pigments, exudates, and colony reversals. The micromorphological features used for characterizing the species were the shapes of conidial heads; the presence or absence of metulae between vesicles and phialides (i.e., uniseriate or biseriate); the color of stipes; and the dimensions, shapes and textures of stipes, vesicles, metulae (when present), phialides, and conidia. To perform micromorphological observations, microscopic mounts were made in lactic acid from MEA colonies after 7 days, and a drop of 70% ethanol was added to remove air bubbles and excess conidia.

### Molecular characterization of sisal endosphere- and root zone soil-associated black aspergilli

Genomic DNA was extracted using the UltraClean® Microbial DNA Isolation kit (Mo Bio, CA, USA). The integrity and quantification of genomic DNA were evaluated by fluorimetry using the Qubit 2.0 Fluorimeter® (Invitrogen). The partial sequence of the CaM gene was amplified with the primers CMD5 (CCGAGTACAAGGARGCCTTC) and CMD6 (CCGATRGAGGTCATRACGTGG) according to Hong et al. (2005). The reactions were prepared in a final volume of 50 μl with the following reagents and concentrations: 60 ng of DNA of each sample, 1 × dAmpliTaq Gold® 360 Master Mix (Life Technologies) and 0.5 pmol/μl of each primer (forward and reverse).

Successfully amplified PCR products were purified using the Illustra® GFX PCR DNA and Gel Band Purification kit (GE Healthcare Life Sciences) and sequenced on an ABI3130 automated sequencer (Applied Biosystems, Life Technologies Q7, CA, USA). The sequences were manually edited using Geneious software (version 9.1.6) (Kearse et al., 2012) and deposited into the NCBI GenBank (Table 1).

### Phylogenetic analyses of sisal endosphere- and root zone soil-associated black aspergilli

The sequences generated in this work were combined with partial CaM sequences of the type specimens of *Aspergillus* section *Nigri* (Samson et al., 2014). The sequence of the type specimen of *Aspergillus carbonarius* was used as the outgroup since the *A. carbonarius* clade is the sister group of the *A. niger* “aggregate” clade. The newly generated sequences of our study (GenBank accession no. MG322269-MG322292) and additional sequences downloaded from GenBank are listed in Table 1 and Supplementary Table 2, respectively. The datasets were aligned using MAFFT v.7 (Katoh and Standley, 2013) under the G-INS–i criteria. Then, they were manually inspected using MEGA v.7 (Tamura et al., 2013). The best-fit model of nucleotide evolution to the datasets was selected by both the Bayesian information criterion (BIC) and the corrected Akaike's information criterion (AICc) using jModelTest2 v.1.7 (Guindon and Gascuel, 2003; Darriba et al., 2012).

All four main methods of phylogenetic analysis, distance (D), maximum parsimony (MP), maximum likelihood (ML), and Bayesian (B), were used to evaluate the dataset. Phylogenetic analyses were performed in PAUP 4.0b10 (Swofford, 2002) and MrBayes 3.2 (Ronquist and Huelsenbeck, 2003).

Bayesian inference (BI) phylogenetic analysis was also applied to the datasets. BI was performed using MrBayes 3.1.2 with two independent runs, each one beginning from random trees with four simultaneous independent chains, performing 1 × 10^7^ replications, sampling one tree every 1 × 10^3^ generations. The first 2.5 × 10^6^ sampled trees were discarded as burn-ins and checked by the convergence criterion (frequencies of the average standard deviation of split <0.01), while the remaining trees were used to reconstruct a 50% majority rule consensus tree and calculate the Bayesian posterior probabilities (BPP) of the clades. A node was considered strongly supported if it showed a BPP ≥ 95% and/or BS ≥ 70%.

### Sequence diversity analyses of sisal endosphere- and root zone soil-associated black aspergilli and comparison with public databases worldwide

Searches for sequence nucleotide variations (SNVs) to identify putative single nucleotide polymorphisms (SNPs) and characterize the haplotypes at the species level of both the sequences from our study (Table 1) and all publicly available CaM sequences from the same identified fungal species around the world (Supplementary Table 3) were performed using Geneious v. 9.1.6 (Kearse et al., 2012). To identify and characterize the SNPs, we used a minimum variant frequency = 0.05 and calculated the *p-*values based on the approximate *p*-value method.

## Results

### Koch's postulates and pathogenicity tests in healthy sisal bulbils under greenhouse conditions and time-course analysis of the infection

**Figure 1:**
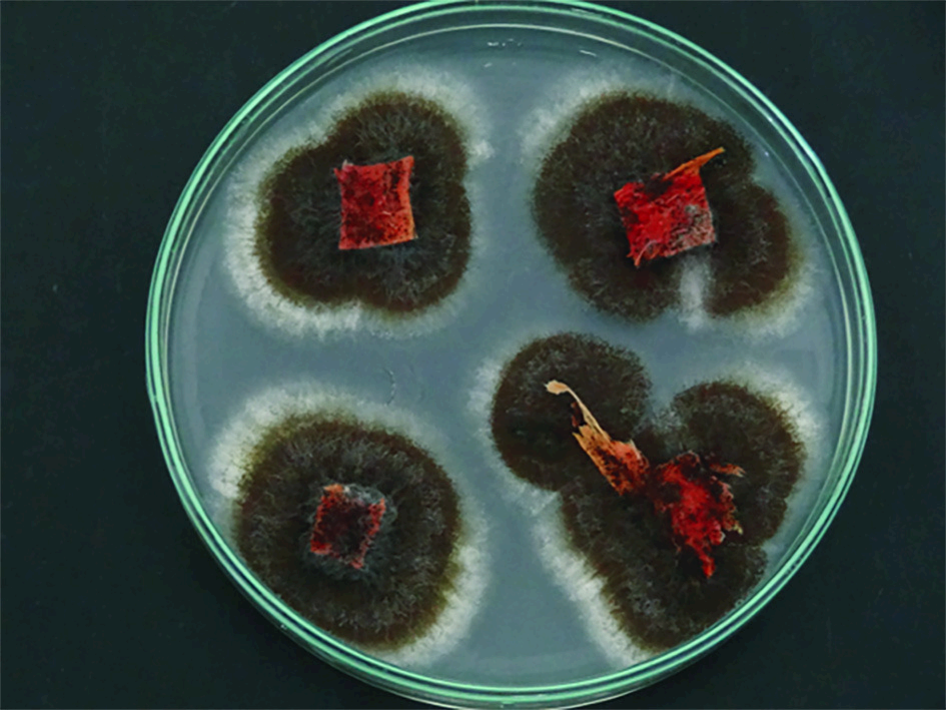
Black aspergilli isolated from the endosphere of symptomatic sisal plants. Black aspergillus mycelia emerging from four explants of the aerial stem (bole) of infected sisal plants inoculated onto culture media in a Petri dish.

**Figure 2:**
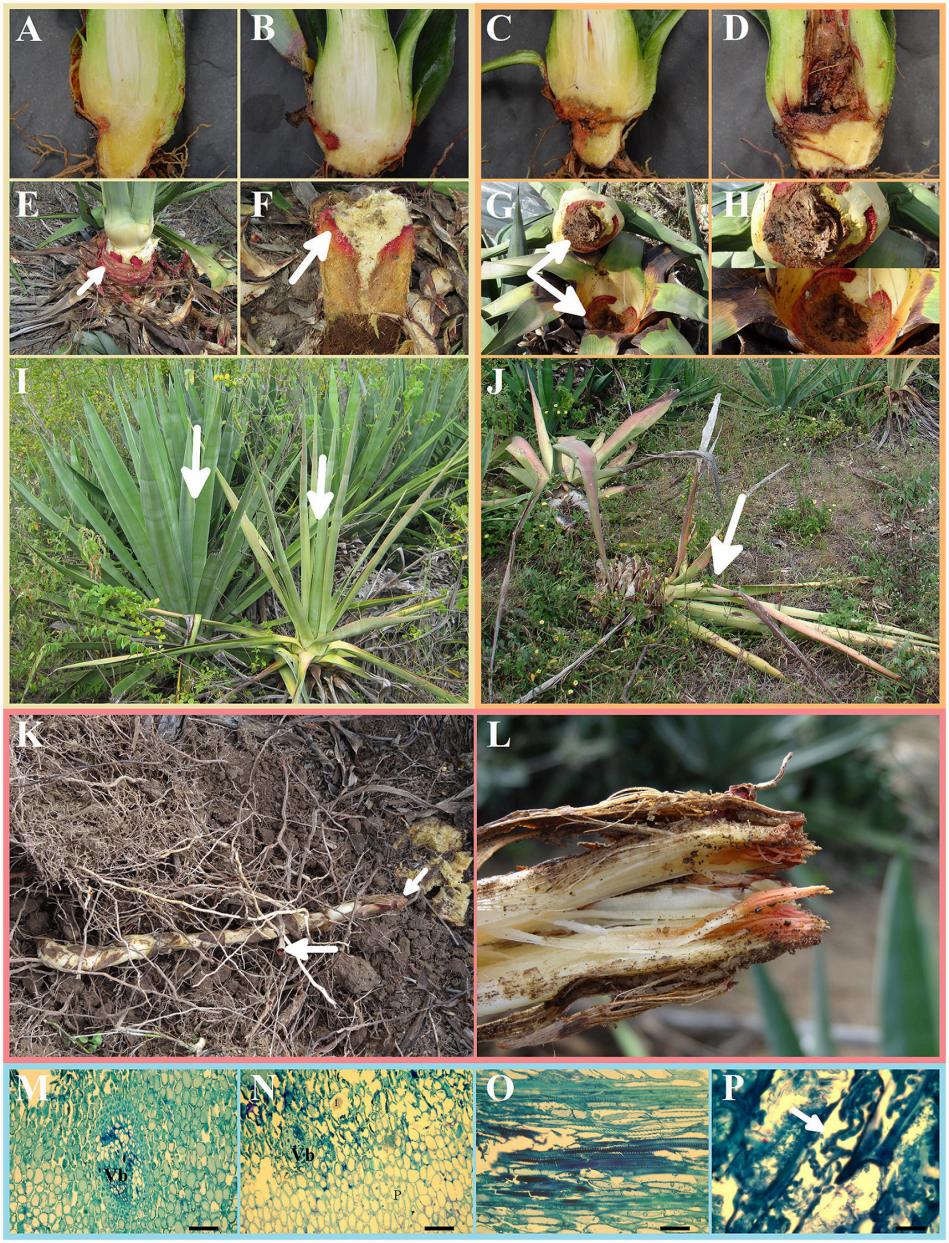
Symptoms of bole rot disease in healthy sisal bulbils grown under greenhouse conditions and adult plants in the field **(I–L)** and histopathology of the infected boles of adult plants **(M–P)**. **(A–D)** Gross longitudinal sections of an artificially infected sisal bulbil showing the internal lesion of the aerial stem. **(E)** External view of an infected aerial stem showing the typical reddish color due to the fungal infection (white arrow). **(F)** Detailed view of the internal infected tissues of the aerial stem (white arrow). **(G,H)** Gross transversal sections in distinct magnifications showing the necrotic lesions of the aerial stem. **(I)** Healthy adult plant (left white arrow) beside a symptomatic adult plant (right white arrow) in the field. **(J)** Dead adult plant due to fungal infection (white arrow). **(K)** An external view of a symptomatic rhizome (subterraneous stem) of an adult plant in the field. **(L)** Gross longitudinal section of a rhizome of an adult plant in the field. **(M,N)** Histological transverse sections of an infected aerial stem of an adult plant, showing a degraded vascular bundle, parenchymatic cells and idioblast. VB, vascular bundle; **(P)** parenchyma; I, idioblast; **(O,P)** histological longitudinal sections of an infected aerial stem of an adult plant at different magnifications, showing fungal hyphae in intercellular spaces and inside parenchymatic cells (white arrow).

All the isolated fungal strains of black aspergilli from the endosphere (Figure 1) and corresponding soil in the root zone of adult infected sisal plants were pathogenic and unequivocally produced the symptoms of bole rot disease in healthy sisal bulbils under greenhouse conditions (Figure 2). The infection began in the bole base, which is the region of the foliar sheaths that is closer to the soil surface and the micro-wounded tissue (Figures 2A,B), and progressed in the direction of the bole apex (Figures 2C,D). The bulbil phenotype comprised initially reddish lesions that became brownish in the center with black fungal conidiophores with progression of the disease (Figures 2A–D). Except for the presence of black fungal sporulation, which did not occur in all plants, the symptoms were the same as those observed under field conditions in adult plants of commercial plantations (Figures 2E–H). The spores from the conidiophores of fungal sporulation were re-inoculated in healthy sisal bulbils and, again, produced the symptoms of bole rot disease. Thus, Koch's postulates were confirmed.

**Figure 3:**
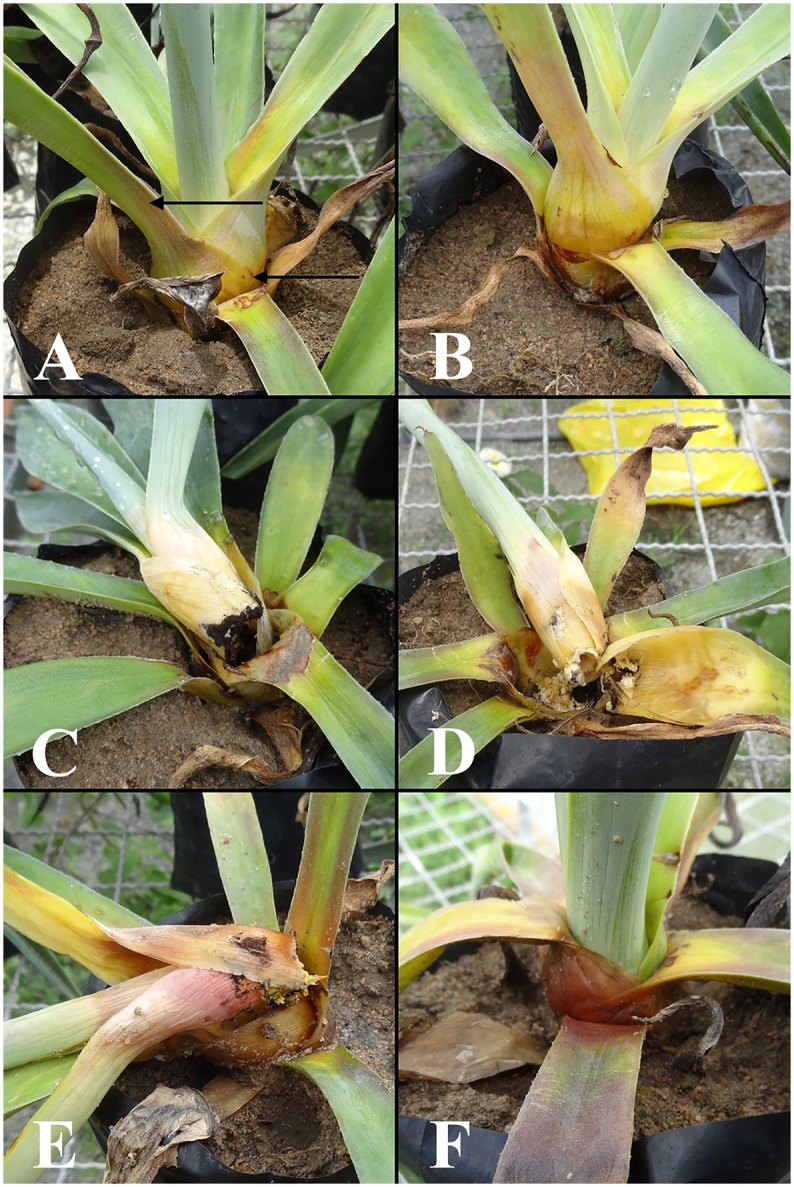
Time-course of the infection in sisal bulbils grown under greenhouse conditions. **(A,B)** External symptoms 8–10 days after the inoculation. The black arrows show chlorosis and wilt. **(C–F)** Aerial stem necrosis and plant death (after 12–15 days).

Usually, the first symptoms were observed within 8–10 days after the inoculation. The base of the leaves showed the external symptoms of chlorosis and wilt, and some leaves began to show a humid rot symptom. In 12–15 days, these symptoms progressed to form bole necrosis but without plant death. After 15 days, some bulbils were dead, and the boles were totally necrotic, with the leaves easily detaching from the bole. In these bulbils, the rot symptoms were humid and yellowish, and the boles became completely necrotic, with black fungal conidiophores on the infected tissues (Figures 3A–D). A typical reddish color in the bole and leaf base along with leaf chlorosis and wilting, bole necrosis with easily detached leaves, and black conidiophores in the infected tissue was also observed in the bulbils 12–15 days after inoculation (Figures 3E,F). These signals of the pathogenic fungus and the symptoms described in Figures 3A–D were more frequent in younger bulbils with thinner boles and softer tissues. In 30 days, most of the bulbils were dead with necrotic boles and yellowish or reddish leaf base symptoms. Only a few of the bulbils survived for more than 30 days.

### Anatomy and histology of the infected boles of sisal adult specimens in the field

All the adult plants analyzed in this sisal-producing area exhibited the same symptoms. The plants with bole rot disease showed typical external symptoms: wilted and chlorotic leaves and, in some plants, reddish and bended leaves (Figure 2I, right arrow), and the healthy plants did not show these symptoms (Figure 2I, left arrow). The internal symptoms were observed as necrosis in the bole leaf base (Figures 2E–H) and rhizomes (Figures 2K,L), all of which exhibited reddish rot tissue. The progression of the disease caused plant death (Figures 2J). Longitudinal and transversal sections of the bole indicated that the disease was disseminated along the ground parenchyma from the cortex to the vascular bundle (Figures 2F–H). The typical reddish color produced by the disease was observed in all the tissues infected by the fungal pathogen (Figures 2F,L).

In the histological sections, the parenchymatic cells of bole tissues infected by the fungal pathogen showed green-stained cell walls stained with toluidine blue, while the cells of healthy tissues were stained blue (Figures 2M–P). In the region affected by the disease, the parenchymatic cells showed degraded walls without a defined shape (Figures 2M,N,P) compared to healthy tissue, whose cells were isodiametric with thin walls. In the bole tissues with red rot, the vascular bundles were completely destroyed, exhibiting degradation of vessel elements of the xylem, and obliteration of their lumina (Figures 2M,N). The longitudinal sections of infected sisal boles displayed the presence of the fungal pathogen inside parenchymatic cells (Figure 2O). Fungal hyphae were also found in intercellular spaces (Figure 2O).

### Morphological characterization and molecular phylogenetic analyses of sisal endosphere- and root zone soil-associated black aspergilli

**Figure 4:**
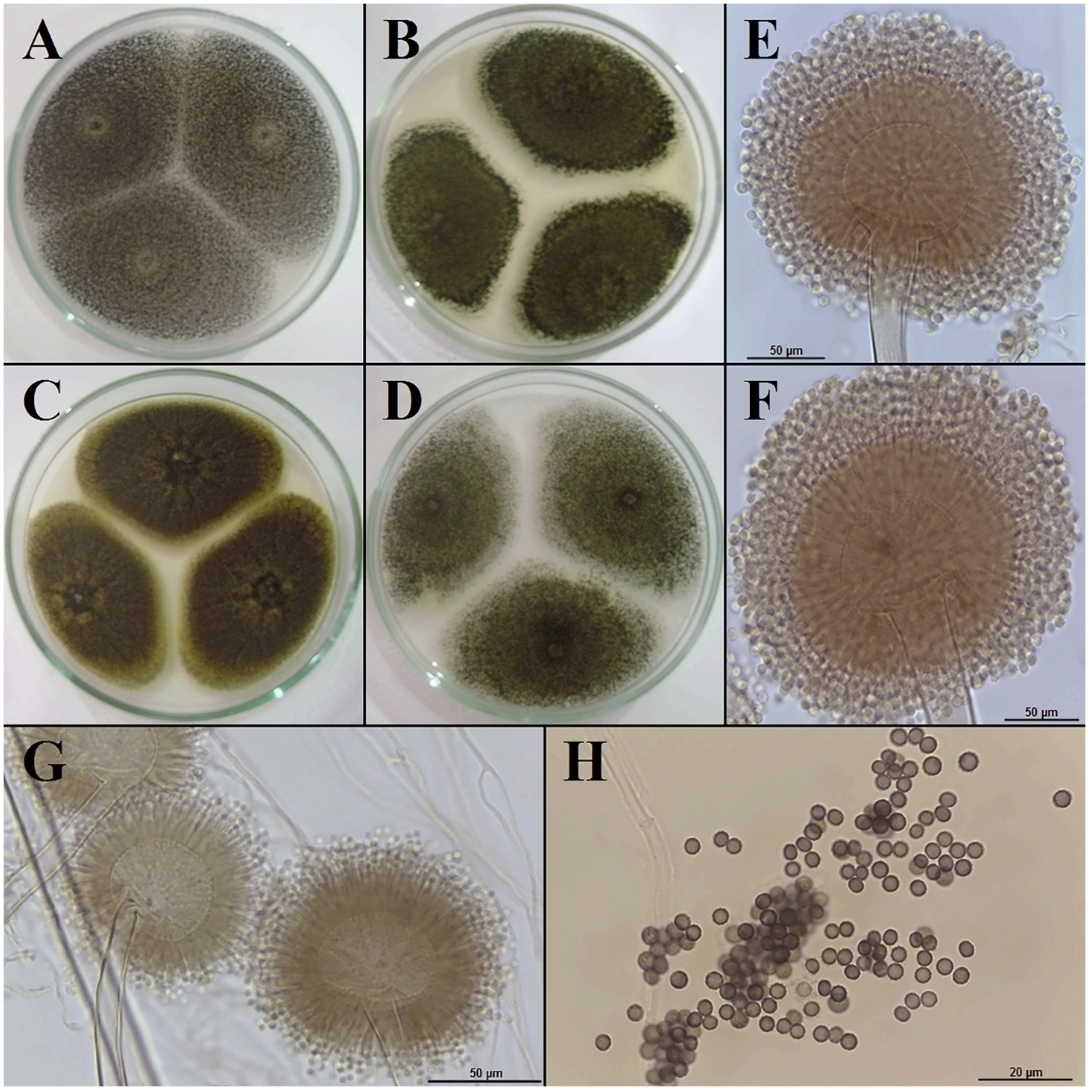
Macro- and micromorphological aspects of black aspergilli isolated from the symptomatic sisal plants. **(A–D)** Aspect of the mycelial growth of colonies in distinct culture media (CYA, MEA, CZA, OA). **(E–G)** Conidiophores. **(H)** Conidia.

All the isolates of sisal endosphere- and root zone soil-associated black aspergilli had overlapping macro- and micromorphological qualitative (shape, ornamentation, etc.) and quantitative (size) characteristics in the mycelia, conidiophores and conidia (Figures 4A–H; Table 2). Similarly, the isolates had similar ranges of growth rates on the media CYA, MEA, and OA, and most had optimum growth temperatures of 25, 28, or 30°C (Table 2).

**Table 2:** Macro- and micromorphological features of plant endosphere- and root zone soil-associated black aspergilli isolated from symptomatic sisal plants in the study areas.

**#**	**Code**	**Colony morphology (CYA for 7 days at 25°C)**	**Colony growth at 7 days (mm) 25°, 30°C and 37°C**			**Conidial head shape**	**Conidiophore**		**Vesicle size (μm)**	**Metulae size (μm)**	**Phialides**		**Conidia**	
			**CYA**	**MEA**	**OA**		**Stipe description**	**Size (μm)**			**Shape**	**Size (μm)**	**Diameter (μm)**	**Shape and ornamentation**
1	RSDI	Black colonies with a white leading edge	40–70	45–70	40–60	Radiated	Biseriate, smooth, and colorless	224–787 × 10–16.5	22.5–65	6.5–16 × 3	Ampuliform	4.7– 10.7 × 3	3–4	Light brown to dark brown, globular, rough to finely rough
2	RSDII	Black colonies with a white to yellow leading edge	16–60	20–70	29–65	Radiated	Biseriate, smooth, and colorless	800–900 × 12.5	40–65	12.5 × 3–5	Ampuliform	7.5 × 3	2.5–5	Dark brown to black, globular, smooth to finely rough
3	RRI	Black colonies with a white to yellow leading edge	40–70	45–70	45–70	Radiated	Biseriate, smooth, and colorless	900–977 × 4.5–17	51–68	10.5–24 × 3	Ampuliform	9–13 × 3	2.5–4	Dark brown to black, globular, smooth to finely rough
4	RRII	Black colonies with a white to yellow leading edge	35–60	25–66	46–70	Radiated	Biseriate, smooth, and colorless	750–900 × 15	40–50	10–12.5 × 3–5	Ampuliform	7.5 × 3–5	3–5	Dark brown to black, globular, rough to finely rough
5	RCI	Black colonies with a white to yellow leading edge	22–58	56–60	50–72	Radiated	Biseriate, smooth, and colorless	500–900 × 15–12.5	30–65	10–12.5 × 5	Ampuliform	5 × 3	3–5	Dark brown to black, globular, rough
6	RCII	Black colonies with a white to yellow leading edge	20–48	20–65	35–60	Radiated	Biseriate, smooth, and colorless	600–900 × 12.5–15	40–65	12.5–15 × 3–5	Ampuliform	5–7.5 × 3	2.5–5	Dark brown to black, globular, rough to finely rough
7	CSDI	Black colonies with a white leading edge	40–70	45–70	45–70	Radiated	Biseriate, smooth, and colorless	400–600 × 15–16	49–61	12–20 × 3	Ampuliform	10–12 × 3	3–4.5	Dark brown to black, globular, rough to finely rough
8	CSDII	Black colonies with a white to yellow leading edge	40–70	45–70	45–70	Radiated	Biseriate, smooth, and colorless	238–584 × 8–16	20–60	6–12.5 × 3	Ampuliform	8–10 × 3–4	3–5	Dark brown to black, globular, rough to finely rough
9	CRI	Black colonies with a white leading edge	40–70	35–70	35–60	Radiated	Biseriate, smooth, and colorless	400–900 × 10	40–60	15–20 × 3	Ampuliform	7–10 × 3	2.5–4	Dark brown to black, globular, rough
10	CRII	Black colonies with a white to yellow leading edge	40–70	35–70	45–65	Radiated	Biseriate, smooth, and colorless	250–1000 × 10	30–55	6.5–11 × 3	Ampuliform	6–10 × 3	3–5	Brown to black, globular, finely rough
11	CCI	Black colonies with a white leading edge	40–70	45–70	45–70	Radiated	Biseriate, smooth, and colorless	620–900 × 10	40	7–16 × 3	Ampuliform	7.5–14 × 3	2–3.5	Light brown to dark brown, globular, rough to finely rough
12	CCII	Black colonies with a white leading edge	15–70	35–70	20–65	Radiated	Biseriate, smooth, and colorless	200–550 × 10	22	7–18 × 6–10	Ampuliform	8.5–12 × 5–6	5	Brown to black, globular, rough to echinulate
13	FSDI	Black colonies with a white to yellow leading edge	18–60	22–55	45–60	Radiated	Biseriate/smooth, and colorless	400–900 × 7.5–10	20–45	13–15 × 3–5	Ampuliform	5–7 × 3–4	3–5	Dark brown to black, globular, rough to finely rough
14	FSDII	Black colonies with a white to yellow leading edge	18–64	22–60	45–60	Radiated	Biseriate/smooth and colorless	400–900 × 7.5–10	20–50	12.5–15 × 3–5	Ampuliform	5–10 × 3	2.5–5	Dark brown to black, globular, rough to finely rough
15	FRI	Black colonies with a white to yellow leading edge	40–70	35–90	35–70	Radiated	Biseriate (uniseriate rare)/smooth and colorless	382–772 × 9–15.5	34–64.5	10–20 × 3	Ampuliform	7–9 × 3	2.5–4	Dark brown, globular, rough to finely rough
16	FRII	Black colonies with a white to yellow leading edge	22–57	20–55	30–52	Radiated	Biseriate/smooth and colorless	400–640 × 10–12.5	22.5–52.5	14 × 3–5	Ampuliform	7 × 3–4	3–4	Dark brown to black, globular, rough
17	FCI	Black colonies with a white to yellow leading edge	45–90	35–80	35–80	Radiated	Biseriate (uniseriate rare)/smooth and colorless	285–500 × 12–14.5	32.5–60.5	15–20 × 3	Ampuliform	8–11 × 3	2.5–3.5	Dark brown to black, globular, rough to finely rough
18	FCII	Black colonies with a white to yellow leading edge	18–60	24–70	38–65	Radiated	Biseriate/smooth and colorless	650–1050 × 10–12.5	30–52.5	10–12.5 × 3	Ampuliform	7.5 × 3	2.5–3	Dark brown to black, globular, rough to finely rough
19	SSDI	Black colonies with a white leading edge	40–70	35–70	38–70	Radiated	Biseriate (uniseriate rare)/smooth and colorless	577–726 × 10–13	27–61	11–12 × 3	Ampuliform	5.8–10.5 × 3	2.5–4	Brown to black, globular, rough to finely rough
20	SSDII	Black colonies with a white to yellow leading edge	25–40	40–50	38–60	Radiated	Biseriate/smooth and colorless	380–660 × 10–12.5	27.5–45	9–10 × 4–5	Ampuliform	6 × 4–5	3–4	Dark brown to black, globular, finely rough
21	SRI	Black colonies with a white leading edge	27–65	41–52	29–70	Radiated	Biseriate/smooth and colorless	600–1000 × 10–12.5	35–57.5	7.5–12,5 × 3	Ampuliform	5–10 × 3	2.5–3	Dark brown to black, globular, smooth to finely rough
22	SRII	Black colonies with a white to yellow leading edge	16–60	20–70	29–65	Radiated	Biseriate/smooth and colorless	800–900 × 12.5	40–65	12.5 × 3–5	Ampuliform	7.5 × 3	2.5–5	Dark brown to black, globular, smooth to finely rough
23	SCI	Black colonies with a white to yellow leading edge	40–70	35–70	38–70	Radiated	Biseriate/smooth and colorless	400–900 × 7.5–10	20–45	13–15 × 3–5	Ampuliform	5–7 × 3–4	3–5	Light brown to dark brown, globular, rough to finely rough
24	SCII	Black colonies with a white to yellow leading edge	35–60	25–66	46–70	Radiated	Biseriate/smooth and colorless	400–600 × 15–16	49–61	12–20 × 3	Ampuliform	10–12 × 3	3–4.5	Light brown to black, globular, rough to finely rough

The matrix of the aligned sequences generated in this work (Table 1) combined with partial CaM sequences of the type specimens of *Aspergillus* section *Nigri* (Supplementary Table 2) for phylogenetic analysis was 271 characters long.

#### Maximum parsimony (MP)

**Figure 5:**
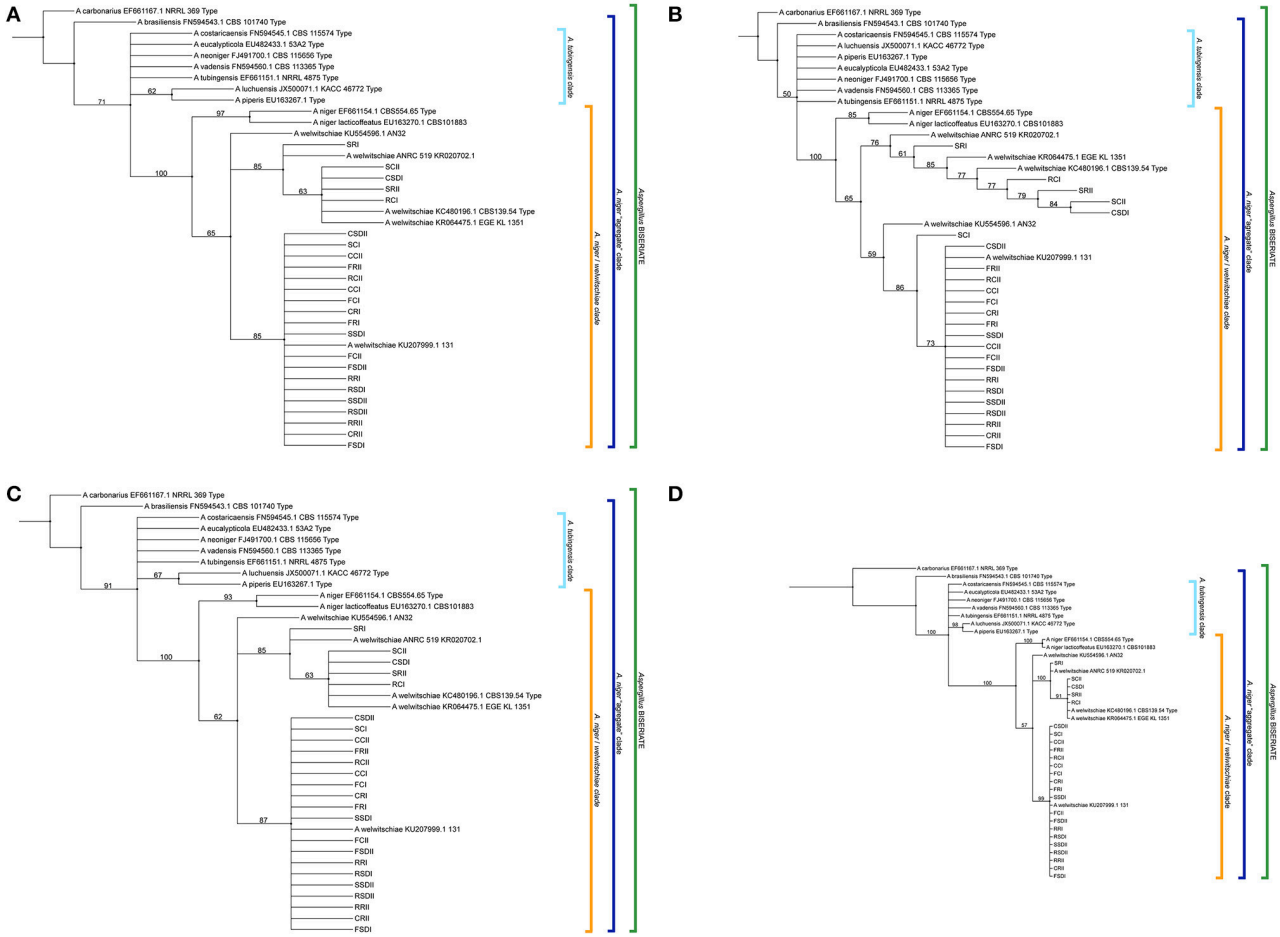
**(A)** Maximum parsimony majority rule consensus tree of *Aspergillus niger* “aggregate” clade type species and black aspergilli isolated from symptomatic sisal plants (bootstrap values above 50% are exhibited). **(B)** Distance tree of *Aspergillus niger* “aggregate” clade type species and black aspergilli isolated from symptomatic sisal plants (bootstrap values above 50% are exhibited). **(C)** Maximum likelihood tree of *Aspergillus niger* “aggregate” clade type species and black aspergilli isolated from symptomatic sisal plants (bootstrap values above 50% are exhibited). **(D)** Bayesian majority rule consensus tree of *Aspergillus niger* “aggregate” clade type species and black aspergilli isolated from symptomatic sisal plants (posterior probability values above 50% are exhibited).

A total of 13.3% of the characteristics were variable, and 91.7% of the variable characteristics were parsimony-informative. The final result of the parsimony analysis comprised the six most parsimonious unrooted trees with 98 evolutionary steps, with a consistency index (CI) = 0.85 and a homoplasy index (HI) = 0.15. After rooting, the majority consensus tree had the following phylogenetic relationships in common: (a) the monophyly of the *A. niger*/*welwitschiae* clade supported by the maximum bootstrap value (BS = 100%), (b) the monophyly of the *A. niger* clade was strongly supported (BS = 92%), and (c) the monophyly of the *A. welwitschiae* clade was moderately supported (BS = 65%). The later clade comprised all the sisal endosphere and root zone soil-associated black aspergilli of our study in addition to the type species and additional *A. welwitschiae* species publicly deposited (Figure 5A). There were also two strongly supported (BS = 85%), less inclusive clades inside the *A. welwitschiae* clade, one of which encompassed most of the sisal isolates (79.2%) and the other comprised the remaining sisal isolates, including the *A. welwitschiae* type species (Figure 5A).

#### Distance (D)

The distance analysis final results comprised only one unrooted tree. After rooting, like the maximum parsimony analysis, the rooted tree showed the same main phylogenetic relationships among the studied taxa. The *A. niger*/*welwitschiae* clade was again maximally supported (BS = 100%), the *A. niger* clade was strongly supported (BS = 85%), and the *A. welwitschiae* clade, containing all sisal isolates, was moderately supported (BS = 65%). The less inclusive clades inside the *A. welwitschiae* clade retrieved in MP analysis were also present; most of the sisal isolates were in one group, and the same other sisal isolates mentioned above, including the *A. welwitschiae* type species, were in another group (Figure 5B).

#### Maximum likelihood (ML)

The maximum likelihood phylogenetic analysis encompassed only one unrooted tree, which exhibited the exact same topology of the MP majority consensus tree after rooting. The support values were very similar, with a stronger value for the *A. niger* clade (BS = 97%) but the same bootstrap proportions for *A. welwitschiae* clade (BS = 65%) and the less inclusive clades inside it (BS = 85%) (Figure 5C).

#### Bayesian (B)

The rooted majority consensus tree of the Bayesian phylogenetic analysis exhibited a topology that was identical to those of ML and MP. Moreover, the branch support values were also very similar, with minimum differences. The *A. niger* clade received the maximum support value (PP = 100%). The *A. welwitschiae* clade received a slight minor posterior probability proportion (PP = 57%), but the less inclusive clades inside it, comprising the same groups evidenced in the other analyses, exhibited maximum to almost maximum support values (PP = 100% and 99%) (Figure 5D).

All the sequences of the strains of sisal pathogenic black aspergilli formed a moderately supported clade with all *A. welwitschiae*, including the sequence of the type specimen. This clade, retrieved in all phylogenies, was clearly distinct from its sister group, the *A. niger* clade, which was nearly maximally or maximally supported in all the analyses.

### Sequence diversity analyses of sisal endosphere- and root zone soil-associated black aspergilli compared with all the same species identified worldwide

The matrix of the aligned sequences generated in this work (*n* = 24) (Table 1) combined with partial CaM sequences of all publicly available sequences of distinct *A. welwitschiae* isolates around the world (*n* = 102) (Supplementary Table 3) comprised 126 sequences.

A total of 10 distinct haplotypes were identified and characterized (Table 3). The most common haplotype (Hap1) comprised 38.9% of all sequences, including the sequence of the type specimen of *A. welwitschiae* and four sisal isolates. These strains were isolated from all three studied areas (Conceição do Coité, São Domingos, and Retirolândia) and from both plant endospheres (stem and root) and the corresponding soil in the root zone. The other strains of this Hap1 haplotype were isolated in North (Canada and Mexico) and South America (southern Brazil), Africa (Benin, Namibia), Europe (Czech Republic and Romania) and Asia (Turkey, Iran, Saudi Arabia, and China), reflecting wide latitudinal and longitudinal ranges. These strains were isolated from distinct substrates, such as host-associated substrates (plant, animal) or directly from natural (soil) or building environments (indoor dust).

**Table 3:** Haplotypes of all *Aspergillus welwitschiae* isolates worldwide (including the sisal *Aspergillus welwitschiae* of our study).

**Haplotype**	**Number**	**Isolate code**	**Position/variable site**													
			**14**	**30**	**41**	**62**	**74**	**75**	**92**	**95**	**99**	**108**	**128**	**191**	**200**	**212**
			**Transition (C:T)**	**Transition (C:T)**	**Transversion (C:G)**	**Transition (G:A)**	**Transition (G:A)**	**Transition (G:A)**	**Transition (T:C)**	**Transition (G:A)**	**Transversion (T:G)**	**Transition (C:T)**	**Transition (T:C)**	**Transversion (C:A)**	**Transversion (T:C)**	**Transition (C:T)**
			**Autoapomorphy (seq. n. 124)**		**Autoapomorphy (seq. n. 92)**	**Autoapomorphy (seq. n.124)**				**Autoapomorphy (seq. n. 91)**						**Autoapomorphy (seq. n. 111)**
**Hap 1**	1	SCII	C	C	C	G	G	G	T	G	T	C	T	C	T	C
	2	CSDI	C	C	C	G	G	G	T	G	T	C	T	C	T	C
	3	SRII	C	C	C	G	G	G	T	G	T	C	T	C	T	C
	4	RCI	C	C	C	G	G	G	T	G	T	C	T	C	T	C
	5	A_welwitschiae_KR064475.1_EGE-KL-1351	C	C	C	G	G	G	T	G	T	C	T	C	T	C
	6	A_welwitschiae_KR064468.1_EGE-KL-1185	C	C	C	G	G	G	T	G	T	C	T	C	T	C
	7	A_welwitschiae_KR064466.1_EGE-KL-1171	C	C	C	G	G	G	T	G	T	C	T	C	T	C
	8	A_welwitschiae_FR751414.1_CCF < CZE_ 4068	C	C	C	G	G	G	T	G	T	C	T	C	T	C
	9	A_welwitschiae_LK031763.1_TUAa4	C	C	C	G	G	G	T	G	T	C	T	C	T	C
	10	A_welwitschiae_LK031761.1_TUAa36	C	C	C	G	G	G	T	G	T	C	T	C	T	C
	11	A_welwitschiae_LK031760.1_TUAa35	C	C	C	G	G	G	T	G	T	C	T	C	T	C
	12	A_welwitschiae_LK031758.1_TUAa33	C	C	C	G	G	G	T	G	T	C	T	C	T	C
	13	A_welwitschiae_LK031757.1_TUAa32	C	C	C	G	G	G	T	G	T	C	T	C	T	C
	14	A_welwitschiae_LK031752.1_TUAa28	C	C	C	G	G	G	T	G	T	C	T	C	T	C
	15	A_welwitschiae_LK031750.1_TUAa26	C	C	C	G	G	G	T	G	T	C	T	C	T	C
	16	A_welwitschiae_LK031749.1_TUAa25	C	C	C	G	G	G	T	G	T	C	T	C	T	C
	17	A_welwitschiae_LK031744.1_TUAa20	C	C	C	G	G	G	T	G	T	C	T	C	T	C
	18	A_welwitschiae_LK031743.1_TUAa2	C	C	C	G	G	G	T	G	T	C	T	C	T	C
	19	A_welwitschiae_LK031739.1_TUAa16	C	C	C	G	G	G	T	G	T	C	T	C	T	C
	20	A_welwitschiae_LK031736.1_TUAa13	C	C	C	G	G	G	T	G	T	C	T	C	T	C
	21	A_welwitschiae_KJ775286.1_DTO_180A9	C	C	C	G	G	G	T	G	T	C	T	C	T	C
	22	A_welwitschiae_KU681064.1_DTO265-G2	C	C	C	G	G	G	T	G	T	C	T	C	T	C
	23	A_welwitschiae_KX894585.1_KAS	C	C	C	G	G	G	T	G	T	C	T	C	T	C
	24	A_welwitschiae_KX894584.1_KAS	C	C	C	G	G	G	T	G	T	C	T	C	T	C
	25	A_welwitschiae_KJ775332.1_DTO_247F7	C	C	C	G	G	G	T	G	T	C	T	C	T	C
	26	A_welwitschiae_LT558746.1_CCF_4963	C	C	C	G	G	G	T	G	T	C	T	C	T	C
	27	A_welwitschiae_KR064473.1_EGE-KL-1318	C	C	C	G	G	G	T	G	T	C	T	C	T	C
	28	A_welwitschiae_KX894578.1_KAS_5970	C	C	C	G	G	G	T	G	T	C	T	C	T	C
	29	A_welwitschiae_KC480196.1_CBS_139.54_TYPE	C	C	C	G	G	G	T	G	T	C	T	C	T	C
	30	A_welwitschiae_KR064484.1_EGE-KL-T-37	C	C	C	G	G	G	T	G	T	C	T	C	T	C
	31	A_welwitschiae_KR064457.1_EGE-KL-1083	C	C	C	G	G	G	T	G	T	C	T	C	T	C
	32	A_welwitschiae_KR064467.1_EGE-KL-1176	C	C	C	G	G	G	T	G	T	C	T	C	T	C
	33	A_welwitschiae_KR064483.1_EGE-KL-T-36	C	C	C	G	G	G	T	G	T	C	T	C	T	C
	34	A_welwitschiae_KX769858.1_LYL7	C	C	C	G	G	G	T	G	T	C	T	C	T	C
	35	A_welwitschiae_KR064479.1_EGE-KL-1399	C	C	C	G	G	G	T	G	T	C	T	C	T	C
	36	A_welwitschiae_KR064481.1_EGE-KL-1451	C	C	C	G	G	G	T	G	T	C	T	C	T	C
	37	A_welwitschiae_LC000569.1_PW3171	C	C	C	G	G	G	T	G	T	C	T	C	T	C
	38	A_welwitschiae_KR064462.1_EGE-KL-1116	C	C	C	G	G	G	T	G	T	C	T	C	T	C
	39	A_welwitschiae_KR064476.1_EGE-KL-1356	C	C	C	G	G	G	T	G	T	C	T	C	T	C
	40	A_welwitschiae_KR064459.1_EGE-KL-1102	C	C	C	G	G	G	T	G	T	C	T	C	T	C
	41	A_welwitschiae_KR064469.1_EGE-KL-1198	C	C	C	G	G	G	T	G	T	C	T	C	T	C
	42	A_welwitschiae_KR064460.1_EGE-KL-1108	C	C	C	G	G	G	T	G	T	C	T	C	T	C
	43	A_welwitschiae_KR064477.1_EGE-KL-1373	C	C	C	G	G	G	T	G	T	C	T	C	T	C
	44	A_welwitschiae_KR064471.1_EGE-KL-1296	C	C	C	G	G	G	T	G	T	C	T	C	T	C
	45	A_welwitschiae_KR064458.1_EGE-KL-1094	C	C	C	G	G	G	T	G	T	C	T	C	T	C
	46	A_welwitschiae_KR064480.1_EGE-KL-1410	C	C	C	G	G	G	T	G	T	C	T	C	T	C
	47	A_welwitschiae_KR064472.1_EGE-KL-1308	C	C	C	G	G	G	T	G	T	C	T	C	T	C
	48	A_welwitschiae_KT749950.1_ITAL_25.132	C	C	C	G	G	G	T	G	T	C	T	C	T	C
	49	A_welwitschiae_KR064463.1_EGE-KL-1146	C	C	C	G	G	G	T	G	T	C	T	C	T	C
**Hap 2**	50	SRI	C	T	C	G	G	G	T	G	T	C	T	C	T	C
	51	A_welwitschiae_KR020702.1_ANRC_519	C	T	C	G	G	G	T	G	T	C	T	C	T	C
	52	A_welwitschiae_LK031768.1_TUAa9	C	T	C	G	G	G	T	G	T	C	T	C	T	C
	53	A_welwitschiae_LK031767.1_TUAa8	C	T	C	G	G	G	T	G	T	C	T	C	T	C
	54	A_welwitschiae_LK031762.1_TUAa37	C	T	C	G	G	G	T	G	T	C	T	C	T	C
	55	A_welwitschiae_LK031759.1_TUAa34	C	T	C	G	G	G	T	G	T	C	T	C	T	C
	56	A_welwitschiae_LK031756.1_TUAa31	C	T	C	G	G	G	T	G	T	C	T	C	T	C
	57	A_welwitschiae_LK031754.1_TUAa3	C	T	C	G	G	G	T	G	T	C	T	C	T	C
	58	A_welwitschiae_LK031753.1_TUAa29	C	T	C	G	G	G	T	G	T	C	T	C	T	C
	59	A_welwitschiae_LK031751.1_TUAa27	C	T	C	G	G	G	T	G	T	C	T	C	T	C
	60	A_welwitschiae_LK031748.1_TUAa24	C	T	C	G	G	G	T	G	T	C	T	C	T	C
	61	A_welwitschiae_LK031747.1_TUAa23	C	T	C	G	G	G	T	G	T	C	T	C	T	C
	62	A_welwitschiae_LK031746.1_TUAa22	C	T	C	G	G	G	T	G	T	C	T	C	T	C
	63	A_welwitschiae_LK031745.1_TUAa21	C	T	C	G	G	G	T	G	T	C	T	C	T	C
	64	A_welwitschiae_LK031742.1_TUAa19	C	T	C	G	G	G	T	G	T	C	T	C	T	C
	65	A_welwitschiae_LK031741.1_TUAa18	C	T	C	G	G	G	T	G	T	C	T	C	T	C
	66	A_welwitschiae_LK031740.1_TUAa17	C	T	C	G	G	G	T	G	T	C	T	C	T	C
	67	A_welwitschiae_LK031738.1_TUAa15	C	T	C	G	G	G	T	G	T	C	T	C	T	C
	68	A_welwitschiae_LK031737.1_TUAa14	C	T	C	G	G	G	T	G	T	C	T	C	T	C
	69	A_welwitschiae_LK031735.1_TUAa12	C	T	C	G	G	G	T	G	T	C	T	C	T	C
	70	A_welwitschiae_LK031734.1_TUAa11	C	T	C	G	G	G	T	G	T	C	T	C	T	C
	71	A_welwitschiae_LK031733.1_TUAa10	C	T	C	G	G	G	T	G	T	C	T	C	T	C
	72	A_welwitschiae_KR020701.1_ANRC_521	C	T	C	G	G	G	T	G	T	C	T	C	T	C
	73	A_welwitschiae_KJ775268.1_DTO_178C2	C	T	C	G	G	G	T	G	T	C	T	C	T	C
	74	A_welwitschiae_FR751417.1_CCF < CZE_ 641	C	T	C	G	G	G	T	G	T	C	T	C	T	C
	75	A_welwitschiae_LN890507.1_FCBP087	C	T	C	G	G	G	T	G	T	C	T	C	T	C
	76	A_welwitschiae_LT558747.1_S776	C	T	C	G	G	G	T	G	T	C	T	C	T	C
	77	A_welwitschiae_KR064482.1_EGE-KL-CD-202	C	T	C	G	G	G	T	G	T	C	T	C	T	C
	78	A_welwitschiae_KR064464.1_EGE-KL-1150	C	T	C	G	G	G	T	G	T	C	T	C	T	C
	79	A_welwitschiae_KR064478.1_EGE-KL-1398	C	T	C	G	G	G	T	G	T	C	T	C	T	C
	80	A_welwitschiae_LC000559.1_PW3050	C	T	C	G	G	G	T	G	T	C	T	C	T	C
	81	A_welwitschiae_LC000561.1_PW3162	C	T	C	G	G	G	T	G	T	C	T	C	T	C
	82	A_welwitschiae_KR064474.1_EGE-KL-1350	C	T	C	G	G	G	T	G	T	C	T	C	T	C
	83	A_welwitschiae_KR064470.1_EGE-KL-1227	C	T	C	G	G	G	T	G	T	C	T	C	T	C
	84	A_welwitschiae_KR064465.1_EGE-KL-1154	C	T	C	G	G	G	T	G	T	C	T	C	T	C
	85	A_welwitschiae_KT749951.1_ITAL_684	C	T	C	G	G	G	T	G	T	C	T	C	T	C
	86	A_welwitschiae_KT749956.1_ITAL_15.931	C	T	C	G	G	G	T	G	T	C	T	C	T	C
**Hap 3**	87	A_welwitschiae_LK031766.1_TUAa7	C	T	C	G	G	G	T	G	T	C	C	C	T	C
	88	A_welwitschiae_LK031765.1_TUAa6	C	T	C	G	G	G	T	G	T	C	C	C	T	C
	89	A_welwitschiae_LK031764.1_TUAa5	C	T	C	G	G	G	T	G	T	C	C	C	T	C
	90	A_welwitschiae_KT749955.1_ITAL_361	C	T	C	G	G	G	T	G	T	C	C	C	T	C
**Hap 4**	91	A_welwitschiae_FR751443.1_CCF < CZE_ 4067	C	C	C	G	G	G	T	A	T	C	T	C	T	C
**Hap 5**	92	A_welwitschiae_KR064461.1_EGE-KL-1112	C	T	G	G	G	G	T	G	T	C	T	C	T	T
**Hap 6**	93	SCI	C	T	C	G	A	A	C	G	G	C	T	C	T	C
	94	CCII	C	T	C	G	A	A	C	G	G	C	T	C	T	C
	95	FRII	C	T	C	G	A	A	C	G	G	C	T	C	T	C
	96	RCII	C	T	C	G	A	A	C	G	G	C	T	C	T	C
	97	CCI	C	T	C	G	A	A	C	G	G	C	T	C	T	C
	98	FCI	C	T	C	G	A	A	C	G	G	C	T	C	T	C
	99	CRI	C	T	C	G	A	A	C	G	G	C	T	C	T	C
	100	FRI	C	T	C	G	A	A	C	G	G	C	T	C	T	C
	101	SSDI	C	T	C	G	A	A	C	G	G	C	T	C	T	C
	102	CSDII	C	T	C	G	A	A	C	G	G	C	T	C	T	C
	103	FCII	C	T	C	G	A	A	C	G	G	C	T	C	T	C
	104	FSDII	C	T	C	G	A	A	C	G	G	C	T	C	T	C
	105	RRI	C	T	C	G	A	A	C	G	G	C	T	C	T	C
	106	RSDI	C	T	C	G	A	A	C	G	G	C	T	C	T	C
	107	SSDII	C	T	C	G	A	A	C	G	G	C	T	C	T	C
	108	RSDII	C	T	C	G	A	A	C	G	G	C	T	C	T	C
	109	RRII	C	T	C	G	A	A	C	G	G	C	T	C	T	C
	110	CRII	C	T	C	G	A	A	C	G	G	C	T	C	T	C
	111	FSDI	C	T	C	G	A	A	C	G	G	C	T	C	T	C
	112	A_welwitschiae_KU207999.1_131	C	T	C	G	A	A	C	G	G	C	T	C	T	C
**Hap 7**	113	A_welwitschiae_KU554596.1_AN32	C	T	C	G	G	G	C	G	G	C	T	C	T	C
	114	A_welwitschiae_KU554595.1_AN12	C	T	C	G	G	G	C	G	G	C	T	C	T	C
	115	A_welwitschiae_KU554594.1_AN7	C	T	C	G	G	G	C	G	G	C	T	C	T	C
	116	A_welwitschiae_KP330148.1_DTO 266-G5	C	T	C	G	G	G	C	G	G	C	T	C	T	C
	117	A_welwitschiae_KP330147.1_DTO 266-E3	C	T	C	G	G	G	C	G	G	C	T	C	T	C
	118	A_welwitschiae_KJ775363.1_DTO_267G1	C	T	C	G	G	G	C	G	G	C	T	C	T	C
	119	A_welwitschiae_KJ775336.1_DTO_266D4	C	T	C	G	G	G	C	G	G	C	T	C	T	C
	120	A_welwitschiae_KJ775333.1_DTO_247G5	C	T	C	G	G	G	C	G	G	C	T	C	T	C
	121	A_welwitschiae_LK031755.1_TUAa30	C	T	C	G	G	G	C	G	G	C	T	C	T	C
	122	A_welwitschiae_KT749952.1_ITAL_47.514	C	T	C	G	G	G	C	G	G	C	T	C	T	C
**Hap 8**	123	A_welwitschiae_LK031732.1_TUAa1	C	T	C	G	G	G	C	G	G	C	T	C	C	C
**Hap 9**	124	A_welwitschiae_KT749953.1_ITAL_48.544	T	T	C	A	G	G	C	G	G	C	T	C	T	C
**Hap 10**	125	A_welwitschiae_KP330149.1_DTO 267-I3	C	T	C	G	G	G	C	G	G	T	T	A	T	C
	126	A_welwitschiae_KT749954.1_ITAL_987	C	T	C	G	G	G	C	G	G	T	T	A	T	C

The second most common haplotype (Hap2) included 29.4% of the sequences and only one sisal isolate (SR1), and it was identical to the Hap1 except for one SNP, a Ti (transition C → T) at the 30th position in the symmetric aligned matrix (Table 3). Similar to Hap1, the haplotype Hap2 contained sequences derived from strains isolated on distinct continents, including America (Brazil, Argentina), Africa (South Africa), and Eurasia (Romania, Turkey, Saudi Arabia, Pakistan, and China). Furthermore, the strains were isolated from both host-associated and environmental substrates.

Most of the sisal isolates in our study (79.2%) and a single isolate from the same geographical region and host (sisal) previously deposited in NCBI formed the haplotype Hap6, which encompassed 16% of all *A. welwitschiae* sequences of the partial CaM gene in public databases. Hap6 exhibited five SNPs (four transitions and one transversion) (Table 4), all of which were in intron 2 of the CaM gene.

**Table 4:** Single nucleotide polymorphisms of all *Aspergillus welwitschiae* isolates worldwide (including the sisal *Aspergillus welwitschiae* of our study).

**N°. SNP**	**Nucleotide**	**Min**.	**Max**.	**Length**	**Coverage**	**Polymorphism type**	**Variant frequency**
1	T	30	30	1	126	SNP	60.3%
	C	30	30	1	126	SNP	39.7%
2	G	74	74	1	126	SNP	84.1%
	A	74	74	1	126	SNP	15.9%
3	G	75	75	1	126	SNP	84.1%
	A	75	75	1	126	SNP	15.9%
4	T	92	92	1	126	SNP	73%
	C	92	92	1	126	SNP	27%
5	T	99	99	1	126	SNP	73%
	G	99	99	1	126	SNP	27%

The Hap7, Hap3 and Hap10 haplotypes were restricted to a few sequences (8.0, 3.2, and 1.6%, respectively), and the others, Hap4, Hap5, Hap8, and Hap9, were autapomorphies of single isolates (Table 3).

**Figure 6:**
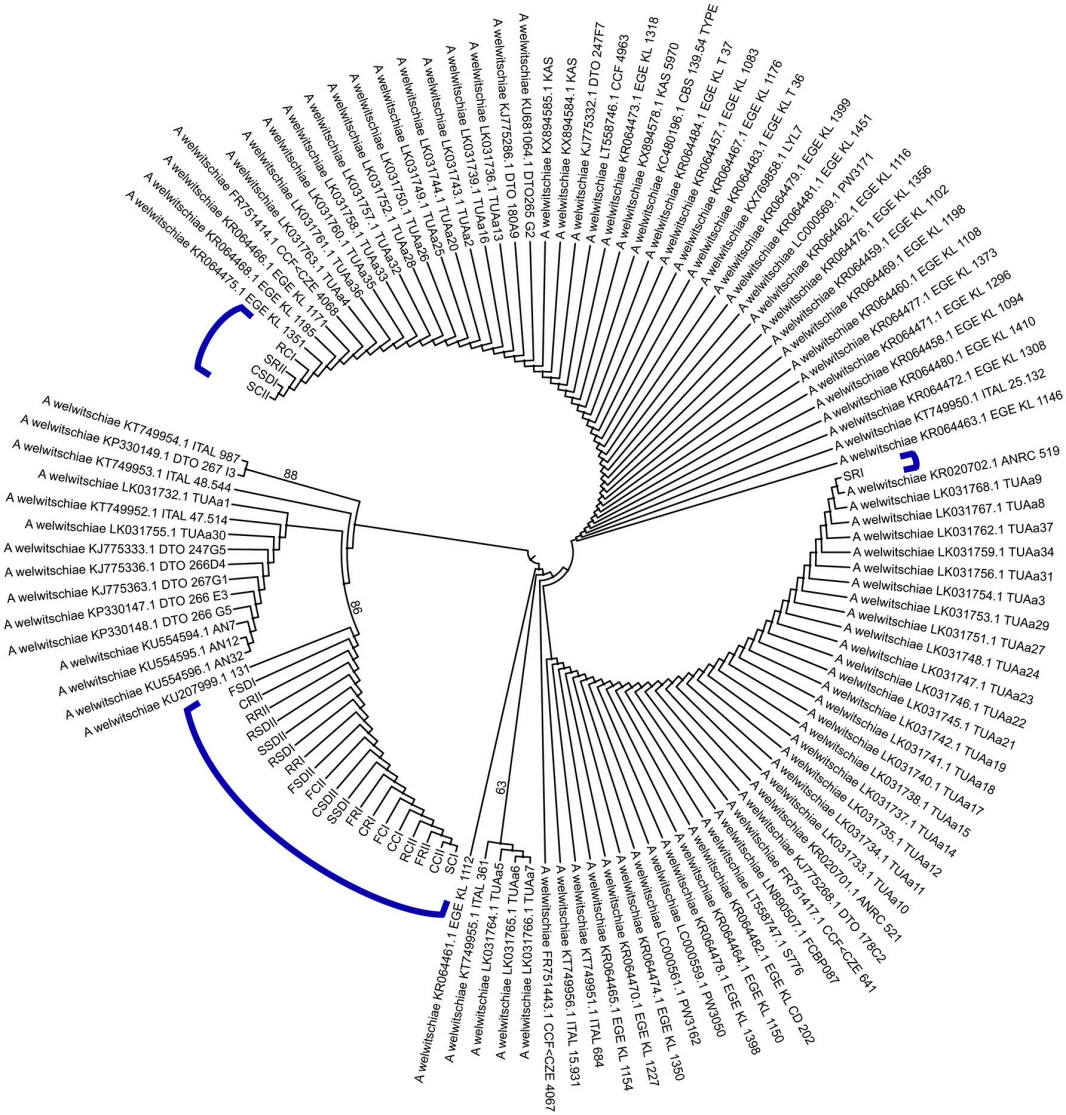
UPGMA tree of worldwide *Aspergillus welwitschiae* (including those *A. welwitschiae* isolated from symptomatic sisal plants in our study).

Therefore, based on the partial CaM gene, at least three different haplotypes (Hap6, Hap1, and Hap2) of *A. welwitschiae* causing sisal bole rot were present in the main sisal-producing area of the world. Figure 6 graphically depicts the intraspecific similarity relationships among all 126 strains of *A. welwitschiae*.

## Discussion

Herein, we performed an integrative analysis of sisal bole rot disease in one of the largest and most important sisal-producing areas of the world. Our study comprised (a) the isolation of black aspergilli in the below- and above-ground endospheres and corresponding soils in the root zones of symptomatic adult sisal plants, (b) *in vivo* pathogenicity tests and time-course analysis of the symptoms of these fungal isolates in bulbils under greenhouse conditions, (c) histopathological analysis of the infected adult plants in field conditions, (d) morphological and molecular characterization and identification and molecular phylogeny analysis of these isolates; and (e) a comparative global-level genetic variability of these molecularly identified isolates with all other known strains.

To determine the relationship of black aspergilli isolated from symptomatic sisal plants with other black aspergilli, we used a hierarchical approach to first define the phylogenetic species and then compare this species with all the publicly deposited sequences on a global scale. In all the tree-based phylogenetic analyses used (distance, maximum parsimony, maximum likelihood and Bayesian) (Figures 5A–D), a monophyletic group comprised by the type species and additional *A. welwitschiae* species and all the sisal endosphere- and root zone soil-associated black aspergilli were retrieved with moderate statistical support inside a more inclusive maximum-supported group representing the *A. niger*/*welwitschiae* clade. Therefore, all four phylogenetic methods were congruent and indicated that the phylogenetic species of the pathogenic sisal isolates is undoubtedly *Aspergillus welwitschiae*. The moderate statistical support for the exclusive *A. welwitschiae* clade in all the analyses suggests that this species recently diverged from *A. niger*. Only a specific study on the calibration and estimation of the divergence time of these two species would confirm this hypothesis (Hedges and Kumar, 2009).

*Aspergillus welwitschiae* was collected for the first time at the end of the nineteenth century in an arid region of southwestern Africa by A. F. Moller from the cone scales of *Welwitschia mirabilis* Hook. f. The material was identified by Bresadola, and the original description was published by Saccardo (1883) as *Ustilago welwitschiae* ßres., n. sp. (page 68). Although the identification was not correct (*Ustilago* is a basidiomycotan genus), the morphological description of the spores (*sporis fuscidulis, globosis, asperulis, 3½ – 4* μ diameter) is in accordance with what is currently known (Table 2). Some years later, Hennings (1903) reclassified *Ustilago welwitschiae* as *Sterigmatomyces welwitschiae*. Finally, Wehmer (1907) reclassified it as *Aspergillus welwitschiae* (Bres.) Henn. apud Wehmer, pointing out that this name was previously proposed by Hennings in a written communication.

Until recently, *Aspergillus welwitschiae* was considered an almost unknown and rare black *Aspergillus* species associated with a very peculiar plant (*Welwitschia mirabilis*) with a restricted geographical distribution (a contiguous region from Angola to Namibia in southwestern Africa). After a detailed study on several species of *Aspergillus* section *Nigri* associated with traditional food fermentations in eastern Asia, Hong et al. (2013) reported that the neotype of *A. awamori* (Perrone et al., 2011) not only did not originate from *awamori* fermentation but was also identical to *Aspergillus welwitschiae*. After the highly clarifying study by Hong et al. (2013), who clearly indicated that *A. welwitschiae* was much more common than believed and explained how to molecularly discriminate *A. welwitschiae* from *A. niger*, several isolates of this species have been reported on distinct substrates and in distinct geographic regions (Supplementary Table 3).

*A. welwitschiae* (designed as *A. niger*) naturally occurs in the reproductive structures of *W. mirabilis*, including cones and seeds (Cooper-Driver et al., 2000; Whitaker et al., 2004, 2008; Pekarek et al., 2006). *Aspergillus welwitschiae* is a common inhabitant of soil (Eicker et al., 1982; Whitaker et al., 2008), and its spores are present in the air (Whitaker et al., 2008) of the arid and semiarid regions where *W. mirabilis* occurs. There is a very high prevalence of infected seeds in natural populations (Whitaker et al., 2008), and most of the newly formed seedlings (at least in *ex situ* studies) die from *A. welwitschiae* infection (Ursem, 2004).

In addition to having been directly associated with its original substrates and a specific geographic distribution, isolates of *A. welwitschiae* have also been reported in fresh or dried fruits, such as almonds (Susca et al., 2016), Brazil nuts (Massi et al., 2016), cashew nuts (Lamboni et al., 2016), grapes/raisins, figures, maize, pistachios, and walnuts (Susca et al., 2016), as well as in bulbs (modified stems), such as onions (Gherbawy et al., 2015) and garlics (Oh et al., 2016), and mustard seeds (Hanif et al., 2016). Furthermore, *Aspergillus welwitschiae* has been detected in different environmental substrates, such as outdoor air (Lee et al., 2016), indoor dust (Visagie et al., 2014), caves (Nováková et al., 2017), sea salts (Biango-Daniels and Hodge, 2018), and even in clinical specimens from the ear canal in humans, causing otomycoses (Szigeti et al., 2012a,b), and in human nails, causing onychomycoses (Tsang et al., 2016). These records are from distinct climates all over the world (Supplementary Table 3). Therefore, *A. welwitschiae* is far from being a rare black aspergillus species and is indeed a morphological cryptic species (Bickford et al., 2007) of the *A. niger* species complex, currently comprising the monophyletic clade *A. niger*/*welwitschiae* (Samson et al., 2014).

*Aspergillus welwitschiae* was isolated in all the soil samples from root zones of all the investigated sites in the semiarid sisal-producing region of Brazil (Table 1). Thus, it may be quite common in the soil of that region and, most likely, opportunistically infects the internal sisal tissues. It was also present inside the tissues of the vegetative organs (roots, stem, and leaves) in the symptomatic plants sampled in three distinct areas (Table 1).

Originally thought be a probable specialized necrotrophic parasite of *W. mirabilis, A. welwitschiae* now appears to be a saprotrophic fungal species that facultatively parasitizes plants using a necrotrophic nutritional mode. To better investigate this possibility, we studied the histopathology of sisal bole rot disease in field symptomatic adult plants for the first time. Based on the histopathological findings and symptomatology, the penetration of the fungus in the host tissues occurred from the exterior (epidermis) to the interior (parenchyma and, subsequently, to the vascular cylinder) as well as from the inferior portion of the bole in the foliar sheath (closer to the soil) to the superior portion of the bole in the apical meristem (Figures 2D,E). As *A. welwitschiae* commonly occurred in the soil around the root zone of adult symptomatic plants (Table 1) and apparently only penetrates sisal tissues via natural or artificial openings (Wallace and Dieckmahns, 1952), damaged foliar sheaths in the inferior portion of the bole near the soil must be the most likely site of fungal penetration into the host, and the histopathological findings corroborate this hypothesis.

Neto and Martins (2012) described the histologies of leaves, rhizomes and roots in adult healthy *A. sisalana* plants in field conditions. The main histological differences between our sampled infected adult plants and healthy adult plants were the cell wall degradation of both the parenchymatic and vascular cylinder cells (Figures 2M,N,P), corroborating the external symptom of leaf wilting. Therefore, in symptomatic sisal plants, *A. welwitschiae* acts as a typical general necrotrophic pathogen, which destroys living cells and feeds on their contents, living saprotrophically on these dead remains.

Similar fungus-plant pathosystems in plant stems occur between soil-borne black aspergilli and peanuts (*Arachis hypogea*) (Gibson, 1953; Moraes, 2006) and *Dracaena sanderiana* (Abbasi and Aliabadi, 2008). However, only the macromorphological external symptoms (yellow-brown lesions extending into the plant tissues) and signals (black conidiophores and conidia) were described without any histopathological description.

After the fungal species that causes sisal bole rot was taxonomically and phylogenetically characterized and defined, we investigated the genetic diversity of *A. welwitschiae* both on a local scale (those isolated from plants of the sisal-producing area) and on a global scale (all the *A. welwitschiae* recorded worldwide). Surprisingly, even using one molecular marker (the partial CaM gene), three distinct haplotypes were identified in sisal isolates from the sisal-producing area of northeastern Brazil from a total of 10 haplotypes identified in all the publicly available sequences around the world (including the sisal isolates). Five SNPs, four transitions and one transversion were identified and confirmed to have rather distinct proportions of the variant types (Table 4). Moreover, the most frequent haplotype of the sisal isolates contained all these SNPs, and it occurs in only the sisal-producing area in Brazil.

Using molecular phylogenetic analyses, our study unequivocally demonstrated that *Aspergillus welwitschiae* (and not *A. niger*) is the causal agent of the sisal bole rot disease. A total of 10 haplotypes of the CaM gene in all *A. welwitschiae* were identified in the entire world, and three of these haplotypes occurred in one of the largest sisal-producing regions of Brazil, which is the highest sisal-producing country worldwide. One of the three haplotypes from sisal isolates (accounting for almost 80% of the total) was present in only sisal plants and the sampled region. All the *Aspergillus welwitschiae* strains isolated from the endospheres (roots/stems/leaves) and corresponding soils in the root zones of adult symptomatic plants in field conditions induced the typical symptomatology in healthy bulbils. Inside the host, the fungus destroyed the parenchymatic and vascular cylinder cells, growing inter- and intracellularly via dead cells, and induced the necrosis of internal stem tissues. Therefore, sisal bole rot disease is the consequence of a saprotrophic fungus that opportunistically invades sisal plants and behaves as a typical necrotrophic pathogen.

Although there have been significant advances in understanding sisal bole rot disease, there still are many open questions regarding the relationships between *Aspergillus welwitschiae* and sisal, such as the role of the mycotoxins in the progression of the disease, the innate and adaptive immune responses of the plant, and the structure and function of the microbiomes in healthy and symptomatic plants. We are currently investigating these questions using an integrative omics approach.

## Author contributions

AG-N, AS, ED, and TdO conceived and designed the experiments. AG-N, AS, ED, CD, LB, FM, TdO, TdL, JdQ, and RdS performed the experiments. AG-N, AS, ED, CD, LB, FM, TdO, TdL, RK, DB, and VA analyzed the data. AG-N, AS, ED, TdO, and VA wrote the paper.

### Conflict of interest statement

The authors declare that the research was conducted in the absence of any commercial or financial relationships that could be construed as a potential conflict of interest.

## Supplementary material

Supplementary Table 1 Soil characteristics of the studied areas.

Supplementary Table 2 *Aspergillus niger* “aggregate” clade type species used in our study.

Supplementary Table 3 Publicly available sequences of *Aspergillus welwitschiae* and their metadata used in our study.
