# Supplementary material for: Putting the Mess in Order: Aspergillus welwitschiae (and Not A. niger) Is the Etiological Agent of Sisal Bole Rot Disease in Brazil
Source: Front Microbiol. 2018 Jun 11;9:1227. doi: 10.3389/fmicb.2018.01227 (PMC6004399; doi:10.3389/fmicb.2018.01227)
Supplement: Supplementary Table 1 — Soil characteristics of the studied areas. [file Table_1.docx]

**Table S1**: Soil characteristics of the study areas.

| **Areas** | **pH** | **P** | **K** | **Ca** | **Mg** | **H+Al** | **S** | **CEC** | **BS** | **SOM** |
| --- | --- | --- | --- | --- | --- | --- | --- | --- | --- | --- |
|  |  | **(mg/dm^3^)** | | **(Cmol_c_/dm^3^)** | | | | | **(%)** | **(g/dm^3^)** |
| São Domingos (S) | 9.45 | 100 | 150 | 14.5 | 4.4 | 1.07 | 20.2 | 21.3 | 94.9 | 11.3 |
| Conceição do Coité (C) | 5.27 | 10 | 47 | 2.2 | 1,4 | 1 | 3.8 | 4.82 | 79.2 | 9.7 |
| Retirolândia (R) | 7.11 | 37 | 69 | 18.7 | 3.7 | 0.94 | 22.6 | 23.5 | 96 | 11.4 |

Methods: pH in CaCl_2_ (active acidity) - CaCl_2_ 0,01 mol.l^-1^; SOM.- Dichromate/Colorimetric; Phosphorus, Potassium, Calcium and Magnesium – Ion exchange resin; H+Al (potential acidity) - pH SMP; Boron – hot water/microwave; Cu, Fe, Mn e Zn - extraction DTPA - TEA in pH 7.3; Na – double acid extraction; Si - extraction CaCl_2_ 0.01 mol.l^-1^.
