# Supplementary material for: Putting the Mess in Order: Aspergillus welwitschiae (and Not A. niger) Is the Etiological Agent of Sisal Bole Rot Disease in Brazil
Source: Front Microbiol. 2018 Jun 11;9:1227. doi: 10.3389/fmicb.2018.01227 (PMC6004399; doi:10.3389/fmicb.2018.01227)
Supplement: Supplementary Table 2 — Aspergillus niger “aggregate” clade type species used in our study. [file Table_2.DOCX]

**Table S2**: *Aspergillus niger* “aggregate” clade type species used in our study.

| Species | Access no. NCBI | TYPE |
| --- | --- | --- |
| Aspergillus welwitschiae | KC480196.1 | Yes |
| Aspergillus welwitschiae | KR064475.1_ | No |
| Aspergillus welwitschiae | KR020702.1 | No |
| Aspergillus welwitschiae | KU207999.1 | No |
| Aspergillus welwitschiae | KU554596.1 | No |
| Aspergillus niger | EF661154.1 | Yes |
| Aspergillus niger (as: Aspergillus lacticoffeatus) | EU163270.1 | No |
| Aspergillus brasiliensis | FN594543.1 | Yes |
| Aspergillus costaricaensis | FN594545.1 | Yes |
| Aspergillus luchuensis | JX500071.1 | Yes |
| Aspergillus piperis | EU163267.1 | Yes |
| Aspergillus eucalypticola | EU482433.1 | Yes |
| Aspergillus neoniger | FJ491700.1 | Yes |
| Aspergillus vadensis | FN594560.1 | Yes |
| Aspergillus tubingensis | EF661151.1 | Yes |
| Aspergillus carbonarius | EF661167.1 | Yes |

___________________________________________________________________
